# Post-Partum Hemorrhage and Its Treatment

**Published:** 1881-02-20

**Authors:** S. P. Sackett

**Affiliations:** N. Y.


### POST-PARTUM HEMORRHAGE AND ITS TREATMENT.

BY S. P. SACKETT, M.D., N. Y.

[Read before the Tompkins County Medical Society.]

As I wish to say something of flooding following delivery, I will commence by relating a case that recently occurred in my practice.

October 26th, 1880, I was called to attend Mrs. Eldridge, aged 25, primipara. Her appearance was anæmic, and she said that she had not previously had good health. I delivered her after a labor of thirty hours' continuance, of a male child weighing nine pounds, using forceps at the last, after I was satisfied that there was inertia of the womb, and that the pains were evidently inefficient.

Before applying the forceps she was laid across the bed, with her feet drawn up on the edge of the bed, and the child and then the after-birth delivered, without any unusual difficulty or hemorrhage, and from the position and exposure of the parts, I was able to observe just how much blood was lost. Immediately afterward the blood came in a profuse torrent, producing some evident change in her pulse and countenance, which showed some signs of approaching collapse.

My hand had been placed on the abdomen, to make pressure while I removed the placenta, and I again placed my left hand on the bowels and endeavored to grasp the uterus, while I passed my right hand into the vagina to reach the womb, to stimulate it to contract, by friction on the inside. At first I removed two clots as large as my fist from the vagina and womb, and held my hand quite still in the vagina. Within about one minute the flooding ceased, and after holding my hand still one minute longer, I removed it, and also the new clots that had formed, and the patient did not suffer from any unusual hemorrhage afterward. She had a tolerably good recovery, though she remained for some time weak and anæmic.

Without commenting on this case further than to say that, in a similar case I should try the plan again of using my hand as a tampon, I will proceed to give my views of the treatment of post partum hemorrhage.

In order to be prepared for an emergency, the practitioner of obstetrics should in each case be armed with ergot, opium, veratrum, some diffusible stimulant, such as aromatic ammonia, aromatic powder or brandy, one of the persalts of iron, tinct. iodine, carbolic acid, permanganate of potash and alum, or aluminate of iron, glycerine, ergotine and acetate of lead. He should also, without parade, see that hot and cold water are near at hand, and, as I think, also vinegar, and a syringe suitable for vaginal and one for hypodermic use, and ice or snow, if obtainable. I have made a long list of articles, because I can think of some contingencies in which I might desire either of them.

Either opium or ergot may be safely and properly given a short time before the termination of labor, as a precaution to prevent hemorrhage. I may say also that I tie the cord in only one place (instead of two and cutting it between the ligatures), and I am confident that I have less trouble (from either adherent placenta or hemorrhage) thereby. The practice, also, of assisting the expulsion of the placenta by pressure and friction on the abdomen while inducing contraction of the uterus, lessens the liability to flooding.

If troublesome hemorrhage does come on, I should not depend, for even a minute, entirely upon such manipulations with my hands as I have indicated. While doing what I could myself, I should direct a nurse or assistant to ligate the thigh tightly with a cord, a practice that has been effective in other hemorrhage, and might be in this.

Subsequent treatment should, of course, vary with the symptoms. If there is faintness or great prostration, I would give an ounce of brandy with nutmeg, believing that it would equalize the circulation and lessen the flow, although I do not permit my patients to take alcoholic stimulants during the labor, lest it should cause flooding. Should the pulse be full or hard, we should administer the following remedies successively or alternately if requried: Ergot, opium, veratrum, and large doses of acetate of lead.

Without waiting for the effect of internal remedies, I would apply a piece of ice or a cloth wet in cold water to the abdomen, and perhaps press a piece of ice into the vagina.

We should not continue to use the cold applications more than half an hour if they are inefficient, and if we do not have ice or snow at hand we may apply a cloth quite hot immediately to the abdomen. At the same time, I would introduce a cloth saturated with vinegar into the uterus, and after squeezing it so as to leave the vinegar there, I would remove the cloth as far down as the vagina. Should hemorrhage still continue, use the following successively as injections into the vagina and womb. 1st. Hot water, of the temperature of 110° to 120°. 2d. A solution of permanganate of potash. 3d. A solution of glycerine and carbolic acid. 4th. A weak solution of iodine. 5th. A solution of some of the salts of iron, of which I prefer Monsel's solution.

I would be willing to substitute alum for either of the above drugs if that was at hand and the others were not. Ergotin may be substituted for ergot, and may be administered hypodermically.

In conclusion, I would say that a pulse of 100 or more, after delivery, indicates danger of hemorrhage, and the patient should be watched. *Medical and Surgical Reporter.*

